# *cis*-Expression QTL Analysis of Established Colorectal Cancer Risk Variants in Colon Tumors and Adjacent Normal Tissue

**DOI:** 10.1371/journal.pone.0030477

**Published:** 2012-02-17

**Authors:** Lenora W. M. Loo, Iona Cheng, Maarit Tiirikainen, Annette Lum-Jones, Ann Seifried, Lucas M. Dunklee, James M. Church, Robert Gryfe, Daniel J. Weisenberger, Robert W. Haile, Steven Gallinger, David J. Duggan, Stephen N. Thibodeau, Graham Casey, Loïc Le Marchand

**Affiliations:** 1 Epidemiology Program, University of Hawaii Cancer Center, Honolulu, Hawaii, United States of America; 2 Department of Colorectal Surgery, Cleveland Clinic Foundation, Cleveland, Ohio, United States of America; 3 Department of Surgery, Samuel Lunenfeld Research Institute, Mount Sinai Hospital, Ontario, Canada; 4 University of Southern California Epigenome Center, Keck School of Medicine, University of Southern California, Los Angeles, California, United States of America; 5 Department of Preventive Medicine, Norris Comprehensive Cancer Center, Keck School of Medicine, University of Southern California, Los Angeles, California, United States of America; 6 Integrated Cancer Genomics Division, Translational Genomics Research Institute, Phoenix, Arizona, United States of America; 7 Department of Laboratory Medicine and Pathology, Mayo Clinic, Rochester, Minnesota, United States of America; Ohio State University Medical Center, United States of America

## Abstract

Genome-wide association studies (GWAS) have identified 19 risk variants associated with colorectal cancer. As most of these risk variants reside outside the coding regions of genes, we conducted *cis*-expression quantitative trait loci (*cis*-eQTL) analyses to investigate possible regulatory functions on the expression of neighboring genes. Forty microsatellite stable and CpG island methylator phenotype-negative colorectal tumors and paired adjacent normal colon tissues were used for genome-wide SNP and gene expression profiling. We found that three risk variants (rs10795668, rs4444235 and rs9929218, using near perfect proxies rs706771, rs11623717 and rs2059252, respectively) were significantly associated (FDR *q*-value ≤0.05) with expression levels of nearby genes (<2 Mb up- or down-stream). We observed an association between the low colorectal cancer risk allele (A) for rs10795668 at 10p14 and increased expression of *ATP5C1* (*q* = 0.024) and between the colorectal cancer high risk allele (C) for rs4444235 at 14q22.2 and increased expression of *DLGAP5* (*q* = 0.041), both in tumor samples. The colorectal cancer low risk allele (A) for rs9929218 at 16q22.1 was associated with a significant decrease in expression of both *NOL3* (*q* = 0.017) and *DDX28* (*q* = 0.046) in the adjacent normal colon tissue samples. Of the four genes, *DLGAP5* and *NOL3* have been previously reported to play a role in colon carcinogenesis and *ATP5C1* and *DDX28* are mitochondrial proteins involved in cellular metabolism and division, respectively. The combination of GWAS findings, prior functional studies, and the *cis*-eQTL analyses described here suggest putative functional activities for three of the colorectal cancer GWAS identified risk loci as regulating the expression of neighboring genes.

## Introduction

Genome-wide association studies (GWAS) of colorectal cancer have revealed 19 common genetic variants at 14 loci that contribute to the risk of colorectal cancer [1], [2], [3], [4], [5], [6], [7]. All but one (rs10936599) of these risk variants reside in intronic, intergenic or gene-desert regions (Table 1) and may serve as markers for causal variants that regulate neighboring or distant genes. Thus, the current challenge is to elucidate how these risk variants specifically influence the development of colorectal cancer. One promising approach is to evaluate these variants for their associations with differential gene expression since transcript abundance may act as a useful intermediate phenotype in deciphering the link between a genetic locus and a clinical phenotype [8].

**Table 1:** Nineteen established CRC risk variants identified by GWAS and their proxies considered in this study.

Reference	Locus	SNP	Position^a^	Closest Gene	Major Allele/Minor Allele^b^	Minor Allele Frequency^c^	Proxy Used	r^2^ d	Proxy Major Allele/Minor Allele^b^
[18] [2]	1q41	rs6691170^e^	220112069	Intergenic	G/T	0.36	rs11579490	0.90	C/A
[18]	1q41	rs6687758^e^	220231571	Intergenic	A/G	0.20	rs6691195	1.00	C/A
[18]	3q26.2	rs10936599^e^	170974795	*MYNN* (exon)	C/T	0.22	rs12638862	0.95	A/G
[22]	8q23.3	rs16892766	117699864	*EIF3H*	A/C	0.07	-	-	-
[21]	8q24.21	rs6983267	128482487	Intergenic	T/G	0.49	-	-	-
[23]	8q24.21	rs10505477	128476625	*ORF DQ515897*	G/A	0.50	-	-	-
[20]	8q24.21	rs7014346	128493974	*POU5FIP1*	G/A	0.37	-	-	-
[23]	9q24.1	rs719725^e^	6355683	Intergenic	A/C	0.50	rs10975552	0.97	T/C
[22]	10p14	rs10795668^e^	8741225	Intergenic	G/A	0.33	rs706771	0.97	G/A
[20]	11q23.1	rs3802842^e^	110676919	LOC120376 (intron)	A/C	0.29	rs3802840	1	G/T
[18]	12q13.13	rs7136702	49166483	Intergenic	C/T	0.35	-	-	-
[18]	12q13.13	rs11169552^e^	49441930	Intergenic	C/T	0.28	rs11169544	1.00	T/C
[19]	14q22.2	rs4444235^e^	53480669	*BMP4*	T/C	0.46	rs11623717	0.93	A/G
[22]	15q13.3	rs4779584	30782048	Intergenic	C/T	0.19	-	-	-
[19]	16q22.1	rs9929218^e^	67378447	*CDH1* (intron)	G/A	0.29	rs2059254	1.00	C/T
[17], [20], [22]	18q21.1	rs4939827^e^	44707461	*SMAD7* (intron)	C/T	0.47	rs7226855	1.00	G/A
[19]	19q13.1	rs10411210^e^^,^ f	38224140	*RHPN2* (intron)	C/T	0.10	-	-	-
[19]	20p12.3	rs961253^e^	6352281	Intergenic	C/A	0.36	rs5005940	1.00	A/T
[18]	20q13.33	rs4925386	60354439	*LAMA5* (intron)	C/T	0.31	-	-	-

a Position based on dbSNP build 130.

b Major allele/minor allele among Europeans.

c Minor allele frequencies from published reports.

d Linkage disequilibrium between SNP and proxy in HapMap CEU.

e Not on Affymetrix 6.0 array.

f Excluded from analysis as proxy r^2^<0.90.

Gene expression levels are highly heritable [9], [10], [11] and differential gene expression can be mapped to a particular genetic locus as an expression quantitative trait locus (eQTL) affecting nearby (*cis-*) or distant (*trans-*) genes [12], [13]. Indeed, GWAS risk loci have been reported to be enriched for eQTLs, providing insight into possible mechanistic effects as well as aiding in the identification of additional variants that can account for the heritability of disease [14]. While several previous eQTL studies have been conducted almost exclusively in lymphoblastoid cell lines [12], [15], [16], a few recent studies have shown tissue-specific associations between genetic variants and gene expression [17], [18], [19]. For cancer risk loci, the eQTL associations observed in the originating tissue giving rise to the tumor are expected to be more informative [20].

To uncover whether established risk variants for colorectal cancer affect expression of neighboring genes differentially by genotype, we conducted a *cis*-eQTL analysis of the GWAS-identified colorectal risk variants using the paired colon adjacent-normal and tumor tissue samples collected from 40 colon cancer patients. This is the first study to conduct a *cis*-eQTL analysis on both adjacent normal and tumor tissue from a homogeneous group of molecularly characterized colorectal tumors (MSS and CIMP-negative).

## Materials and Methods

### Ethics Statement

Approval for this study was obtained in accordance with local Institutional Review Board (IRB) requirements in all participating centers. All subjects included in this study signed an informed written consent.

### Study Subjects and Tissue Samples

Fresh-frozen, colon adenocarcinomas and paired adjacent normal tissue samples were collected at three sites (Mayo Clinic, Mount Sinai, and Cleveland Clinic) from participants in the Colorectal Cancer Family Registry (C-CFR) [21] and tested for microsatellite instability (MSI) and CpG island methylator phenotype (CIMP). Samples from 40 microsatellite stable (MSS)/CIMP-negative tumors, the most common form of colon cancer, and their paired adjacent normal tissue samples (a total of 80 samples) were used for this study. The 40 patients were of European ancestry with an average age of diagnosis of 57 years of age.

### DNA and RNA Isolation

All tumor samples were sectioned and stained with hematoxylin and eosin, then reviewed by a pathologist to determine tumor cell content. Tumor samples used for the study had >70% tumor cell content. Genomic DNA and total RNA were extracted from these tissue samples using the QIAGEN AllPrep DNA/RNA Mini kit (QIAGEN, Valencia, CA) following manufacturer's recommendations.

### MSI and CIMP Testing

MSI status was determined by assaying 10 microsatellite loci (*BAT25*, *BAT26*, *BAT40*, *BAT24C4*, *D5S346*, *D17S250*, *ACTC*, *D18S55*, *D10S197*, and *MYCL*) as previously described [22]. Tumors were classified as MSS if no markers exhibited instability. For CIMP testing, tumor DNA was treated with sodium bisulfite and analyzed using the automated real-time PCR-based MethyLight Assay to identify methylated CpG sites in the promoter regions of an established five-gene panel for CIMP (*CACNA1G*, *IGF2*, *NEUROG1*, *RUNX3*, and *SOCS1*) and in the promoter region of *MLH1*. CIMP status was reported as previously described in [23]. Tumors were classified as CIMP negative if promoter hypermethylation was found in ≤2 genes of the five-gene panel and if there was no *MLH1* promoter DNA methyation.

### Microarray Analysis

Gene expression profiles for colon tumors and adjacent normal tissue were evaluated with the Affymetrix GeneChip Human Exon 1.0 ST Array (Affymetrix, Santa Clara, CA); GEO Accession number GSE31737. Ribosomal RNA (rRNA) was removed from total RNA using the RiboMinus Human/Mouse Transcriptome Isolation Kit (Invitrogen Carlsbad, CA). After rRNA reduction, the Affymetrix GeneChip Whole Transcript (WT) Sense Target Labeling Assay was used to generate amplified and biotinylated sense-strand DNA targets for hybridization on the GeneChip Human Exon 1.0 ST Arrays, following manufacturer's recommendations.

Genomic DNA was extracted from normal colon tissue (n = 34) or blood (n = 6) samples and genotyped using the Affymetrix Genome-Wide Human SNP 6.0 Array. In brief, DNA samples were processed, labeled and hybridized according to the manufacturer's recommendations. All arrays were scanned on The GeneChip® Scanner 3000 7G using the Affymetrix GeneChip Command Console (AGCC) Software to measure the fluorescent signal intensities at each probe location. The average call rate for the 80 samples was 99.6%.

### Selection of risk variants for CRC

We considered all 19 established risk variants for colorectal cancer reported by genome-wide association studies through November, 2010 (Table 1) [1], [2], [3], [4], [5], [6], [7]. Genotype data for 12 of the 19 variants were not available from the Affymetrix 6.0 array (Table 1). For each of these 12 variants not on the array, a proxy was selected among the typed SNPs within a region 20 kb up- or downstream of the risk allele, which was in highest LD (r^2^≥0.90) with the risk variant among HapMap CEU (http://gvs.gs.washington.edu/GVS/). Because rs10411210 at 19q13.1 did not have an acceptable proxy (r^2^<0.90) on the Affymetrix 6.0 array, it was excluded, resulting in a total of 18 risk variants for analysis.

### Real-Time PCR Validation

Technical validation of gene expression profiles was performed on 20 tumor-adjacent normal pairs included in the microarray assays. Real-Time quantitative PCR (qPCR) was conducted for the genes found to be differentially expressed by geneotype in this study (*ATP5C1*, *DLGAP5*, *NOL3*, *DDX28*) and for four genes (*APC*, *MACC1*, *DCC*, and *DSC2*) previously identified to be differentially expressed in colorectal tumors. Briefly, cDNA was prepared from up to 2 µg of untreated total RNA using High Capacity cDNA Reverse Transcription Kit (Applied Biosystems Foster City, CA). For Real-Time qPCR, 21–25 ng of cDNA (based on RNA input) was run on 384-well PCR plates in triplicate using 1× TaqMan gene expression assays and TaqMan Universal PCR Mastermix with the recommended thermal profiles on the 7900HT Fast Real-Time PCR System (Applied Biosystems Foster City, CA).

### Statistical Analysis

For each of the 18 risk variants examined, a *cis*-eQTL analysis was performed to investigate the association between SNP genotypes and gene expression of all nearby genes (within 2 Mb up- and downstream of each SNP). Each SNP was examined by co-dominant and dominant models, using the reported major allele among Europeans as the reference allele. Risk variants having a genotype category with less than 2 samples are not presented. Genome-wide gene expression values were log_2_-transformed and normalized using Robust Multi-array Analysis (RMA), using median polish summarization [24]. The transcript expression value for each gene considered was based on the mean of the probeset intensity for that gene. To identify *cis*-genes associated with differential expression by SNP genotype, multivariate analysis of covariance (ANCOVA) was conducted, adjusting for tumor stage and assay batch. The Benjamini and Hochberg's false discovery rate (FDR) correction was applied to correct for the number of genes tested within the 4 Mb interval surveyed for each risk allele [25]. Spearman rank correlation testing was conducted to validate the correlation between microarray and qPCR assays. The Partek Genomics Suite 6.5 Software (St. Louis, MO) was used for microarray and statistical data analyses.

## Results

In our sample set of 40 paired MSS and CIMP-negative colorectal tumors and adjacent normal tissues, we identified 50 genes that were differentially expressed by genotype for 11 of the 18 risk variants studied (*p*-values <0.05; Table S1). After correcting for multiple-testing, four genes (*ATP5C1*, *DLGAP5*, *NOL3*, *DDX28*) were identified to demonstrate a statistically significant difference in expression levels in the tumor or adjacent normal colon tissue in one or more of the three genotype categories for rs10795668 (10p14), rs4444235 (14q22.2), or rs9929218 (16q22.1) (global test: FDR *q*-value<0.05; Table 2). For rs10795668 at 10p14, we observed a significant difference in gene expression levels by genotype in tumors for the gene encoding for the gamma subunit in the F1 complex of mitochondrial ATP synthase (*ATP5C1*; *q*-value = 0.024). In contrast, there was no difference in gene expression levels by genotype for *ATP5C1* or other neighboring genes in adjacent normal colon tissue for this variant. Similarly, for rs4444235 at 14q22.2, we observed a significant difference in gene expression levels by genotype for the *Drosophila* homolog of discs, large associated protein 5 (*DLGAP5; q*-value = 0.041) when comparing gene expression levels in tumor tissue, but not in adjacent normal tissue. For rs9929218 at 16q22.1, two genes were observed to have a difference in expression levels by genotype: nucleolar protein 3 (*NOL3*; *q*-value = 0.017) and DEAD box polypeptide 28 (*DDX28*, *q*-value = 0.046), in adjacent normal but not tumor tissue.

**Table 2:** Established colorectal cancer risk variants significantly associated with differential gene expression after multiple testing correction.

10p14 rs10795668 (proxy used rs706771)																
			Differential Gene Expression Comparisons^a^													
			All Genotypes													
			(GG = 24; AG = 12; AA = 4)		AG vs. GG (ref)				AA vs. GG (ref)				Any A vs. GG (ref)			
Type of colon tissue	Gene Symbol	Gene Name	p-value	FDR *q*-value	p-value	FDR *q*-value	Relative Expression to Reference	Fold Change	p-value	FDR *q*-value	Relative Expression to Reference	Fold Change	p-value	FDR *q*-value	Relative Expression to Reference	Fold Change
**Normal**	*ATP5C1*	ATP synthase, H+ transporting, mitochondrial F1 complex, gamma polypeptide 1	0.189	0.733	0.993	0.993	up	1.00	0.075	0.120	up	1.21	0.148	0.165	up	1.10
**Tumor**	*ATP5C1*	ATP synthase, H+ transporting, mitochondrial F1 complex, gamma polypeptide 1	**0.005**	**0.024**	0.206	0.926	up	1.07	**0.001**	**0.006**	up	1.36	**0.002**	**0.004**	up	1.21

14q22.2 rs4444235 (proxy used rs11623717)																
			Differential Gene Expression Comparisons^a^													
			All Genotypes													
			(TT = 17; TC = 17; CC = 6)		TC vs. TT (ref)				CC vs. TT (ref)				Any C vs. TT (ref)			
Type of colon tissue	Gene Symbol	Gene	p-value	FDR *q*-value	p-value	FDR *q*-value	Relative Expression to Reference	Fold Change	p-value	FDR *q*-value	Relative Expression to Reference	Fold Change	p-value	FDR *q*-value	Relative Expression to Reference	Fold Change
**Normal**	*DLGAP5*	discs, large (Drosophila) homolog-associated protein 5	0.686	0.971	0.888	0.990	up	1.03	0.412	0.202	up	1.35	0.519	0.210	up	1.18
**Tumor**	*DLGAP5*	discs, large (Drosophila) homolog-associated protein 5	**0.006**	**0.041**	0.012	0.119	up	1.81	**0.003**	**0.014**	up	2.78	**0.002**	**0.032**	up	2.25

16q22 .1 rs9929218 (proxy used rs2059254)																
			Differential Gene Expression Comparisons^a^													
			All Genotypes													
			(GG = 22; GA = 16; AA = 2)		GA vs. GG (ref)				AA vs. GG (ref)				Any A vs. GG (ref)			
Type of colon tissue	Gene Symbol	Gene	p-value	FDR *q*-value	p-value	FDR *q*-value	Relative Expression to Reference	Fold Change	p-value	FDR *q*-value	Relative Expression to Reference	Fold Change	p-value	FDR *q*-value	Relative Expression to Reference	Fold Change
**Normal**	*NOL3*	nucleolar protein 3 (apoptosis repressor with CARD domain)	**2.88×10^−4^**	**0.017**	**9.34×10^−5^**	**0.005**	down	−1.33	0.591	0.933	up	1.08	0.199	0.952	down	−1.11
**Normal**	*DDX28*	DEAD (Asp-Glu-Ala-Asp) box polypeptide 28	**0.002**	**0.046**	**4.15×10^−4^**	**0.012**	down	−1.58	0.917	0.997	up	1.02	0.151	0.952	down	−1.24
**Tumor**	*NOL3*	nucleolar protein 3 (apoptosis repressor with CARD domain)	0.240	0.974	0.179	0.982	down	−1.11	0.458	0.992	up	1.13	0.934	0.992	up	1.04
**Tumor**	*DDX28*	DEAD (Asp-Glu-Ala-Asp) box polypeptide 28	0.249	0.974	0.290	0.982	down	−1.08	0.288	0.992	up	1.17	0.634	0.992	up	1.01

a Based on the alleles of the proxy SNP.

**Figure 1:**
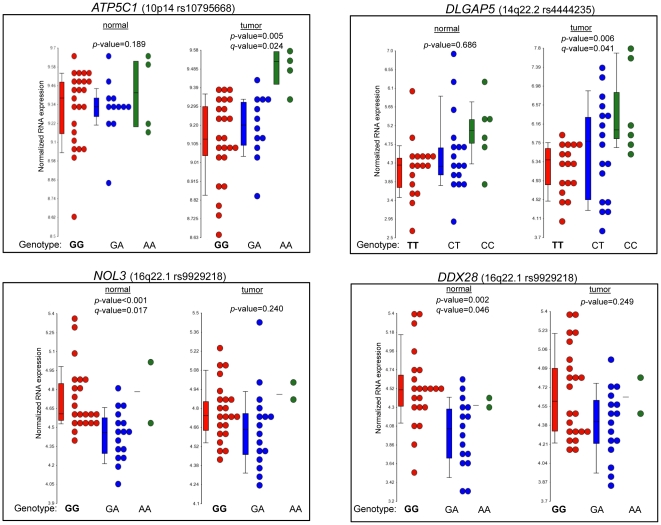
Expression of four genes found to differ by genotype for three colorectal cancer risk variants. Box plots of normalized gene expression levels of *ATP5C1*, *DLGAP5*, *NOL3*, *and DDX28* for paired adjacent normal colon tissue (n = 40) and colon tumor tissue (n = 40). Each point represents the normalized RNA expression levels for an individual. The median gene expression level for each genotype specific group is indicated by a line inside each box within the graph. The *p*-value indicates the significance of the global test comparing expression across genotypes. If the p-values were significant (*p*-value≤0.05), the FDR *q*-values were provided, indicating the significance after correction for multiple comparisons.

The genotype-specific comparisons for the three risk variants with *cis*-eQTL associations are shown in Table 2 and Figure 1. We observed a statistically significant increased expression of *ATP5C1* in the tumors of patients homozygous for the A allele (*q*-value = 0.006) at rs10795668 (10p14) compared to the reference genotype (GG). For rs4444235 (14q22.2), tumors of patients who were homozygous for the C allele had significantly higher expression for *DLGAP5* in comparison to the tumors of those with the reference genotype (TT) (*q*-value = 0.014). For rs9929218 (16q22.1), the genotype specific expression for *NOL3* and *DDX28* in the adjacent normal colon tissue were significantly decreased among patients heterozygous for the A allele versus those with the reference genotype (GG) (*q*-value = 9.34×10^−5^ and *q*-value = 4.15×10^−4^, respectively).

Due to the small number of subjects who were homozygous for the colorectal cancer minor alleles, we also considered gene expression levels in samples that carried either one or two copies of the minor allele, in comparison to the reference genotype (last column of Table 2). Tumor samples from patients with one or two copies of the minor allele(s) (any A) for rs10795668, compared to the GG genotype, demonstrated increased expression of *ATP5C1* at 10p14 (*q*-value = 0.004). Similarly, for the tumor samples of patients with one or two copies of the minor allele(s) (any C) at rs4444235 (14q22.2), expression of *DLGAP5* was increased in comparison to tumors with the TT genotype (*q*-value = 0.032). There was no statistically significant difference in the expression of *NOL3* and *DDX28* in tumor or adjacent normal tissue when comparing patients with one or two copies of the minor allele(s) (A) versus those with the GG genotype for rs9929218 at 16q22.1 (Table 2).

**Figure 2:**
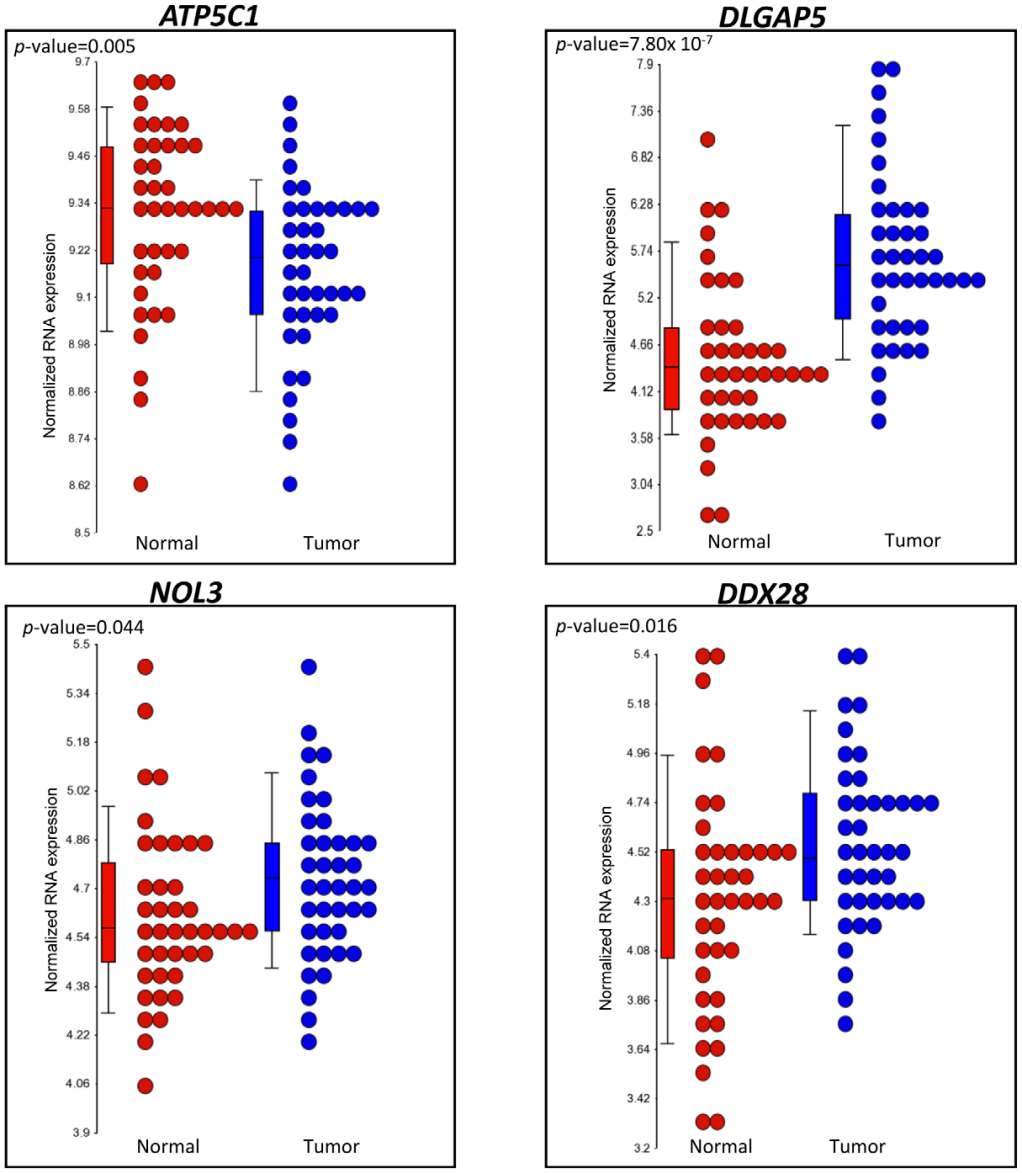
Tumor versus adjacent normal gene expression profiles of the *cis*-eQTL associated genes. Box plots of gene expression levels for *ATP5C1*, *DLGAP5*, *NOL3*, *and DDX28* in paired adjacent normal colon tissue and colon tumor tissue (n = 40 pairs). The significance of differential expression is indicated by the *p*-value.

The four genes that we identified to be differentially expressed in relation to the three risk variants have been shown to have a role in cancer-related mechanisms, such as cellular metabolism and proliferation, and apoptosis [26], [27], [28], [29]. Therefore, we compared the expression levels of the four *cis*-regulated genes (*ATP5C1*, *DLGAP5*, *DDX28*, *NOL3*) between tumors and adjacent normal colon tissue. All four genes were differentially expressed in tumors compared to the adjacent normal colon tissue samples. The expression level of *ATP5C1* (*p*-value = 0.005) was lower in tumors, whereas the expression levels of *DLGAP5* (*p*-value = 7.80×10^−7^), *DDX28* (*p*-value = 0.016) and *NOL3* (*p*-value 0.044) were higher in tumors compared to adjacent normal colon tissue (Figure 2).

To confirm the reliability of the microarray results, we conducted a technical validation using qPCR testing of gene expression levels on 20 cases with remaining RNA, out of the 40 original cases, for both adjacent normal and tumor tissue samples. The Spearman's Rank Order correlation coefficients for the four genes identified in the *cis*-eQTL analysis in the tissue type (tumor or normal) where genotype-specific differential expression was observed were, *ATP5C1 r_s_* = 0.39; *DLGAP5 r_s_* = 0.68; *NOL3 r_s_* = 0.11; *DDX28 r_s_* = 0.22. As an additional technical validation step, we assayed four genes (*APC*, *MACC1*, *DCC*, and *DSC2*) that have been previously established to be differentially expressed between tumor and adjacent normal tissue in colorectal cancer [30], [31], [32], [33]. We found good correlation (Spearman's Rank Order correlation, *r_s_*>0.5) in the gene expression profiles for all four genes between our microarray and qPCR assays. Specifically, lower expression of *APC*, *DCC*, and *DSC2* and higher expression of *MACC1* was observed in the tumor samples relative to the paired adjacent normal tissue in both the microarray and qPCR assays. These technical validation data support the reliability of our observations based on the gene expression microarray results.

## Discussion

Our study examined 18 of the 19 GWAS-identified colorectal cancer risk variants for association with the expression of neighboring genes (within 2 Mb up- and downstream of the SNP) in 40 patients with MSS and CIMP-negative colon cancer, using fresh-frozen paired adjacent normal and colon tumor samples (Figure S1). We identified four genes (*ATP5C1*, *DLGAP5*, *NOL3*, and *DDX28*) at three risk loci with a statistically significant difference in gene expression levels by genotype.

*ATP5C1* encodes the gamma subunit of the catalytic core (F1) of the mitochondrial ATP synthase, the enzyme complex responsible for ATP synthesis, known to play a central role in cellular respiration. A common event in tumor cells is the metabolic switch from respiration (in the mitochondria) to glycolysis (in the cytosol), often referred as “the Warburg effect” [34], [35]. Multiple mechanisms may initiate this switch, one of which is a decrease in the expression of the beta subunit of ATP synthase (F1) (*ATP5B*), leading to the disruption of the catalytic function of the ATP synthase complex, an event that has been previously observed in multiple cancer types [26], [29]. In the tumor samples analyzed in the present study, we observed an increase in the expression levels of *ATP5C1* that was significantly associated with the A allele at rs10795668. The A allele has been associated with a decreased risk of colorectal cancer (OR = 0.89; *p* = 2.5×10^−13^) in a previous GWAS [6]. Thus, the increased expression of *ATP5C1* associated with the A allele would be consistent with maintaining the activities of ATP synthase and cellular respiration and potentially inhibiting tumor progression for colorectal cancer.

*DLGAP5*, also known as *HURP* (hepatoma up-regulated protein), encodes a microtubule binding protein involved in the formation and function of mitotic spindles [36], [37] and is believed to be a cell cycle regulator and target of the Aurora A kinase [38], [39]. Over-expression of *DLGAP5* has been associated with the deregulation of spindle fiber formation and function during mitosis [38]. In addition, it has been reported that *DLGAP5* may have a role in stem cell maintenance and survival and has been observed to be over-expressed in colorectal cancer cells [27], [28], [40]. The C allele for rs4444235 has been previously reported in GWAS to increase the risk of colorectal cancer (OR = 1.11; *p* = 8.1×10^−10^) [3]. Our finding that the C allele is associated with increased *DLGAP5* expression in tumors, suggests a potential mechanism by which this allele may promote tumor progression for colorectal cancer. In addition, we note that rs4444235 has been shown to have a significantly stronger association with MSS-subtypes of colorectal cancer [3], which was the molecular subtype of the tumor samples included in our study.

*DDX28* encodes for a DEAD box protein with RNA helicase activity. Although *DDX28* has not specifically been reported to have a role in colorectal cancer, other DEAD box RNA helicases have been shown to be overexpressed in colorectal tumors, demonstrating a function for RNA helicases in tumorigenesis [41], [42], [43]. The *NOL3* gene, also known as ARC (apoptosis repressor with caspase recruitment domain) encodes for an anti-apoptotic protein that regulates p53 and caspases 2 and 8 [44], [45]. Several studies have shown that *NOL3* is regulated by activated N- and H- Ras and is overexpressed in colorectal cancer [41], [46], [47]. We also observed *NOL3* to be overexpressed in the tumor versus adjacent normal tissue. In addition, our *cis*-eQTL analysis indicated an association for decreased expression of both *DDX28* and *NOL3* in adjacent normal colon tissues of individuals carrying the A allele, particularly cases with the GA genotype for rs9929218. Taken together with the finding that the A allele at rs9929218 is associated with decreased risk of colorectal cancer (OR = 0.91; *p* = 1.2×10^−8^) [3], our observation of an association between this allele and decreased *DDX28* and *NOL3* expression in adjacent normal tissue suggests that these genes may lower risk of colorectal cancers by functioning to inhibit early events of colorectal carcinogenesis. Our findings may also underline the importance of studying normal tissue, in addition to tumor tissue, in eQTL studies of cancer.

Interestingly, the differentially expressed genes identified in this study were not genes directly neighboring the GWAS risk variants. For example, for rs10795668, *GATA3* is the closest neighboring gene, but we did not observe any significant association for differential expression of *GATA3* and this SNP's genotype (*p*-value>0.05). Similarly, for rs4444235 and *BMP4*, which are separated by less than 10 kb, and for rs9929218 which is located in an intron of *CDH1*, we observed no association with genotype and gene expression. In a recent study, Carvajal-Carmona et al. reported on a fine mapping study to colorectal cancer risk alleles at 8q23.3 and 16q22.1 [48]. They also found no association with gene expression of the nearest gene, such as *EIF3H* and *CDH1* in monocyte cell lines, respectively, but find an association with more distant genes such as *UTP23* for rs16892766 at 8q23.3 and *ZFP90* for rs2059254 at 16q22.1 [48]. In our study of colon tissue samples, we also observed an association (*p* = 0.03) for *ZFP90* expression levels and the risk allele at 16q22.1 (Table S1); however the association was no longer significant after adjustments for multiple comparisons. These results, and those of other studies [3], [12], [15], [49], suggest that risk variants may not preferentially regulate genes that are closest. Rather, transcriptional regulatory mechanisms impacted by allelic status may involve complex chromatin confirmation states and function within a tissue specific context.

Few other studies have examined the relationship between colorectal cancer risk variants and gene expression in near-by genes. The COGENT study investigated six GWAS risk variants for their effect on the expression of a small number of neighboring candidate genes in 90 CEU Hapmap, EBV-transformed lymphoblastoid cell lines (rs9929218 for *CDH1* and *CDH3*, rs4444235 for *BMP4*, rs10411210 for *RHPN2*, rs961253 for *BMP2*, rs6983267 for *c-MYC*, and rs3802842 for *LOC120376*) [3]. No significant associations were found. Although we were not able to evaluate rs10411210, we similarly found no associations between the remaining four variants and differential expression of these genes before or after correction for multiple testing in both adjacent normal colon and tumor tissue (*p*-values>0.05). In addition, similar to our findings, a previous study of rs6983267 found no association with *c-MYC* expression in 117 samples of normal colon tissue [50].

This is the most comprehensive and one of the largest tissue-specific *cis*-eQTL studies reported to date for colorectal cancer. Nonetheless, the interpretation of our results is constrained by our limited statistical power (<60% to detect a 15% difference in expression across genotypes) and the need for replication studies with larger sample sizes to confirm the effect of these risk variants on regulating gene expression of neighboring genes. The most notable strengths of this study were the inclusion of both adjacent normal and malignant tissue and the restriction to a homogeneous group of molecularly characterized colorectal tumors (MSS and CIMP-negative).

In summary, our data indicate that the analysis of the effects of risk alleles on gene expression in well-characterized tumors and their paired adjacent normal tissue is likely to be highly informative. Further examination of the risk variants and differentially expressed genes will need to be carried out to confirm our results, as well as expanding the analysis to other molecular subtypes of colorectal cancer and addressing mechanistic events in a tissue specific context.

## Supporting Information

Figure S1 **Flow chart of** ***cis*** **-eQTL analysis of colorectal cancer risk variants.** The flow chart outlines the procedures to analyze of the effects of risk alleles on gene expression, of genes within a 4 Mb range of the risk allele, in well-characterized colorectal tumors and their paired adjacent normal tissue.
(TIF)

Table S1 **Differentially expressed genes associated with risk variants for colorectal cancer.** A list of the 50 genes that were identified to be differentially expressed by genotype for 11 of the 18 risk variants studied (*p*-values<0.05) in the analysis of 40 paired MSS and CIMP-negative colorectal tumor and adjacent normal tissues.
(PDF)

## References

[1] Broderick P, Carvajal-Carmona L, Pittman AM, Webb E, Howarth K (2007). A genome-wide association study shows that common alleles of SMAD7 influence colorectal cancer risk.. Nat Genet.

[2] Houlston RS, Cheadle J, Dobbins SE, Tenesa A, Jones AM (2010). Meta-analysis of three genome-wide association studies identifies susceptibility loci for colorectal cancer at 1q41, 3q26.2, 12q13.13 and 20q13.33.. Nat Genet.

[3] Houlston RS, Webb E, Broderick P, Pittman AM, Di Bernardo MC (2008). Meta-analysis of genome-wide association data identifies four new susceptibility loci for colorectal cancer.. Nat Genet.

[4] Tenesa A, Farrington SM, Prendergast JG, Porteous ME, Walker M (2008). Genome-wide association scan identifies a colorectal cancer susceptibility locus on 11q23 and replicates risk loci at 8q24 and 18q21.. Nat Genet.

[5] Tomlinson I, Webb E, Carvajal-Carmona L, Broderick P, Kemp Z (2007). A genome-wide association scan of tag SNPs identifies a susceptibility variant for colorectal cancer at 8q24.21.. Nat Genet.

[6] Tomlinson IP, Webb E, Carvajal-Carmona L, Broderick P, Howarth K (2008). A genome-wide association study identifies colorectal cancer susceptibility loci on chromosomes 10p14 and 8q23.3.. Nat Genet.

[7] Zanke BW, Greenwood CM, Rangrej J, Kustra R, Tenesa A (2007). Genome-wide association scan identifies a colorectal cancer susceptibility locus on chromosome 8q24.. Nat Genet.

[8] Schadt EE, Lamb J, Yang X, Zhu J, Edwards S (2005). An integrative genomics approach to infer causal associations between gene expression and disease.. Nat Genet.

[9] Cheung VG, Jen KY, Weber T, Morley M, Devlin JL (2003). Genetics of quantitative variation in human gene expression.. Cold Spring Harb Symp Quant Biol.

[10] Morley M, Molony CM, Weber TM, Devlin JL, Ewens KG (2004). Genetic analysis of genome-wide variation in human gene expression.. Nature.

[11] Schadt EE, Monks SA, Drake TA, Lusis AJ, Che N (2003). Genetics of gene expression surveyed in maize, mouse and man.. Nature.

[12] Duan S, Huang RS, Zhang W, Bleibel WK, Roe CA (2008). Genetic architecture of transcript-level variation in humans.. Am J Hum Genet.

[13] Stranger BE, Forrest MS, Clark AG, Minichiello MJ, Deutsch S (2005). Genome-wide associations of gene expression variation in humans.. PLoS Genet.

[14] Nicolae DL, Gamazon E, Zhang W, Duan S, Dolan ME (2010). Trait-associated SNPs are more likely to be eQTLs: annotation to enhance discovery from GWAS.. PLoS Genet.

[15] Dixon AL, Liang L, Moffatt MF, Chen W, Heath S (2007). A genome-wide association study of global gene expression.. Nat Genet.

[16] Veyrieras JB, Kudaravalli S, Kim SY, Dermitzakis ET, Gilad Y (2008). High-resolution mapping of expression-QTLs yields insight into human gene regulation.. PLoS Genet.

[17] Musunuru K, Strong A, Frank-Kamenetsky M, Lee NE, Ahfeldt T (2010). From noncoding variant to phenotype via SORT1 at the 1p13 cholesterol locus.. Nature.

[18] Pomerantz MM, Shrestha Y, Flavin RJ, Regan MM, Penney KL (2010). Analysis of the 10q11 cancer risk locus implicates MSMB and NCOA4 in human prostate tumorigenesis.. PLoS Genet.

[19] Schadt EE, Molony C, Chudin E, Hao K, Yang X (2008). Mapping the genetic architecture of gene expression in human liver.. PLoS Biol.

[20] Freedman ML, Monteiro AN, Gayther SA, Coetzee GA, Risch A (2011). Principles for the post-GWAS functional characterization of cancer risk loci.. Nat Genet.

[21] Newcomb PA, Baron J, Cotterchio M, Gallinger S, Grove J (2007). Colon Cancer Family Registry: an international resource for studies of the genetic epidemiology of colon cancer.. Cancer Epidemiol Biomarkers Prev.

[22] Lindor NM, Burgart LJ, Leontovich O, Goldberg RM, Cunningham JM (2002). Immunohistochemistry versus microsatellite instability testing in phenotyping colorectal tumors.. J Clin Oncol.

[23] Weisenberger DJ, Siegmund KD, Campan M, Young J, Long TI (2006). CpG island methylator phenotype underlies sporadic microsatellite instability and is tightly associated with BRAF mutation in colorectal cancer.. Nat Genet.

[24] Irizarry RA, Hobbs B, Collin F, Beazer-Barclay YD, Antonellis KJ (2003). Exploration, normalization, and summaries of high density oligonucleotide array probe level data.. Biostatistics.

[25] Benjamini Y, Hochber Y (1995). Controlling the false discovery rate: a practical and powerful approach to multiple testing.. Journal of the Royal Statistical Society Series B (Methodological).

[26] Cuezva JM, Krajewska M, de Heredia ML, Krajewski S, Santamaria G (2002). The bioenergetic signature of cancer: a marker of tumor progression.. Cancer Res.

[27] Gudmundsson KO, Thorsteinsson L, Sigurjonsson OE, Keller JR, Olafsson K (2007). Gene expression analysis of hematopoietic progenitor cells identifies Dlg7 as a potential stem cell gene.. Stem Cells.

[28] Tsou AP, Yang CW, Huang CY, Yu RC, Lee YC (2003). Identification of a novel cell cycle regulated gene, HURP, overexpressed in human hepatocellular carcinoma.. Oncogene.

[29] Willers IM, Isidoro A, Ortega AD, Fernandez PL, Cuezva JM (2010). Selective inhibition of beta-F1-ATPase mRNA translation in human tumours.. Biochem J.

[30] Fearon ER, Cho KR, Nigro JM, Kern SE, Simons JW (1990). Identification of a chromosome 18q gene that is altered in colorectal cancers.. Science.

[31] Funakoshi S, Ezaki T, Kong J, Guo RJ, Lynch JP (2008). Repression of the desmocollin 2 gene expression in human colon cancer cells is relieved by the homeodomain transcription factors Cdx1 and Cdx2.. Mol Cancer Res.

[32] Powell SM, Zilz N, Beazer-Barclay Y, Bryan TM, Hamilton SR (1992). APC mutations occur early during colorectal tumorigenesis.. Nature.

[33] Stein U, Walther W, Arlt F, Schwabe H, Smith J (2009). MACC1, a newly identified key regulator of HGF-MET signaling, predicts colon cancer metastasis.. Nat Med.

[34] Warburg O (1930). The Metabolism of Tumors.

[35] Levine AJ, Puzio-Kuter AM (2010). The control of the metabolic switch in cancers by oncogenes and tumor suppressor genes.. Science.

[36] Koffa MD, Casanova CM, Santarella R, Kocher T, Wilm M (2006). HURP is part of a Ran-dependent complex involved in spindle formation.. Curr Biol.

[37] Wong J, Fang G (2006). HURP controls spindle dynamics to promote proper interkinetochore tension and efficient kinetochore capture.. J Cell Biol.

[38] Wong J, Lerrigo R, Jang CY, Fang G (2008). Aurora A regulates the activity of HURP by controlling the accessibility of its microtubule-binding domain.. Mol Biol Cell.

[39] Yu CT, Hsu JM, Lee YC, Tsou AP, Chou CK (2005). Phosphorylation and stabilization of HURP by Aurora-A: implication of HURP as a transforming target of Aurora-A.. Mol Cell Biol.

[40] Bassal S, Nomura N, Venter D, Brand K, McKay MJ (2001). Characterization of a novel human cell-cycle-regulated homologue of Drosophila dlg1.. Genomics.

[41] Lin F, Wang R, Shen JJ, Wang X, Gao P (2008). Knockdown of RCK/p54 expression by RNAi inhibits proliferation of human colorectal cancer cells in vitro and in vivo.. Cancer Biol Ther.

[42] Nakagawa Y, Morikawa H, Hirata I, Shiozaki M, Matsumoto A (1999). Overexpression of rck/p54, a DEAD box protein, in human colorectal tumours.. Br J Cancer.

[43] Shin S, Rossow KL, Grande JP, Janknecht R (2007). Involvement of RNA helicases p68 and p72 in colon cancer.. Cancer Res.

[44] Foo RS, Nam YJ, Ostreicher MJ, Metzl MD, Whelan RS (2007). Regulation of p53 tetramerization and nuclear export by ARC.. Proc Natl Acad Sci U S A.

[45] Heikaus S, Kempf T, Mahotka C, Gabbert HE, Ramp U (2008). Caspase-8 and its inhibitors in RCCs in vivo: the prominent role of ARC.. Apoptosis.

[46] Mercier I, Vuolo M, Jasmin JF, Medina CM, Williams M (2008). ARC (apoptosis repressor with caspase recruitment domain) is a novel marker of human colon cancer.. Cell Cycle.

[47] Wu L, Nam YJ, Kung G, Crow MT, Kitsis RN (2010). Induction of the apoptosis inhibitor ARC by Ras in human cancers.. J Biol Chem.

[48] Carvajal-Carmona LG, Cazier JB, Jones AM, Howarth K, Broderick P (2011). Fine-mapping of colorectal cancer susceptibility loci at 8q23.3, 16q22.1 and 19q13.11: refinement of association signals and use of in silico analysis to suggest functional variation and unexpected candidate target genes.. Hum Mol Genet.

[49] Stranger BE, Forrest MS, Dunning M, Ingle CE, Beazley C (2007). Relative impact of nucleotide and copy number variation on gene expression phenotypes.. Science.

[50] Prokunina-Olsson L, Hall JL (2009). No effect of cancer-associated SNP rs6983267 in the 8q24 region on co-expression of MYC and TCF7L2 in normal colon tissue.. Mol Cancer.

