# Supplementary figures and images for: cis-Expression QTL Analysis of Established Colorectal Cancer Risk Variants in Colon Tumors and Adjacent Normal Tissue
Source: PLoS One. 2012 Feb 17;7(2):e30477. doi: 10.1371/journal.pone.0030477 (PMC3281844; doi:10.1371/journal.pone.0030477)

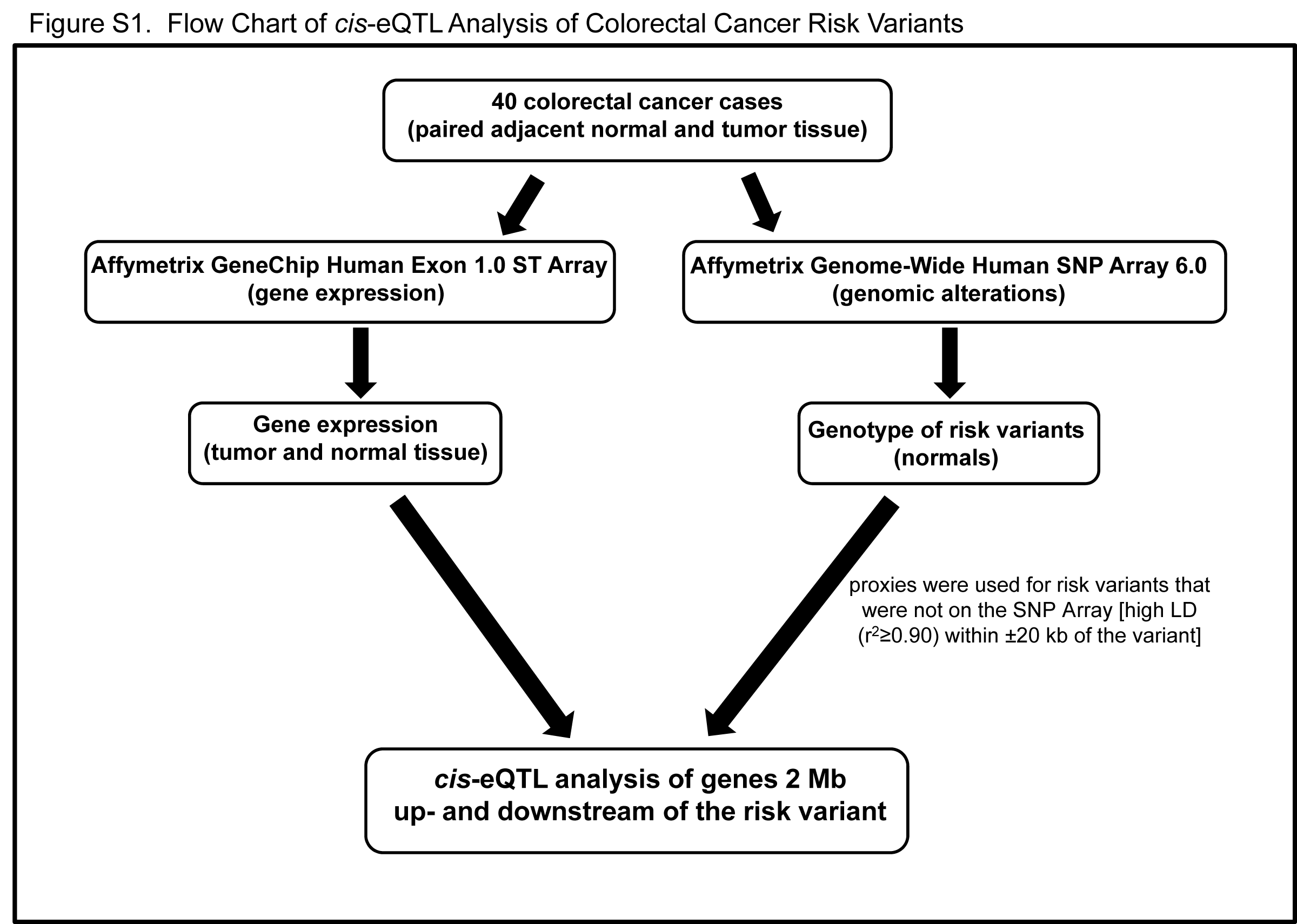

Supplement: Figure S1 — Flow chart of cis -eQTL analysis of colorectal cancer risk variants. The flow chart outlines the procedures to analyze of the effects of risk alleles on gene expression, of genes within a 4 Mb range of the risk allele, in well-characterized colorectal tumors and their paired adjacent normal tissue. (TIF) [file pone.0030477.s001.tif]
