# Supplementary material for: cis-Expression QTL Analysis of Established Colorectal Cancer Risk Variants in Colon Tumors and Adjacent Normal Tissue
Source: PLoS One. 2012 Feb 17;7(2):e30477. doi: 10.1371/journal.pone.0030477 (PMC3281844; doi:10.1371/journal.pone.0030477)
Supplement: Table S1 — Differentially expressed genes associated with risk variants for colorectal cancer. A list of the 50 genes that were identified to be differentially expressed by genotype for 11 of the 18 risk variants studied (p-values<0.05) in the analysis of 40 paired MSS and CIMP-negative colorectal tumor and adjacent normal tissues. (PDF) [file pone.0030477.s002.pdf]

Table S1. Differentially expressed genes nomimally associated (*p* -value < 0.05) with established risk variants for colorectal cancer

SNP	Locus	Tumor/Normal	RefSeq	Gene Symbol	Gene	*p* -value
rs6687758	1q41	Tumor	NM_005681	*TAF1A*	TATA box binding protein (TBP)-associated factor, RNA polymerase I, A	0.043
rs6687758	1q41	Normal	NM_022831	*AIDA*	axin interactor, dorsalization associated	0.020
rs6687758	1q41	Tumor	NM_003268	*TLR5*	toll-like receptor 5	0.037
rs10936599	3q26	Normal	NM_001122752	*SERPINI1*	serpin peptidase inhibitor, clade I (neuroserpin)	0.007
rs10936599	3q26	Normal	NM_014498	*GOLIM4*	golgi integral membrane protein 4	0.024
rs10936599	3q26	Normal	NM_005602	*CLDN11*	claudin 11	0.012
rs10936599	3q26	Normal	NM_020949	*SLC7A14*	solute carrier family 7	0.003
rs719725	9q24	Normal	NM_203453	*PPAPDC2*	phosphatidic acid phosphatase type 2 domain containing 2	0.018
rs719725	9q24	Normal	NM_014143	*CD274*	CD274 molecule	0.020
rs719725	9q24	Normal	NM_025239	*PDCD1LG2*	programmed cell death 1 ligand 2	0.019
rs719725	9q24	Tumor	NM_017913	*CDC37L1*	cell division cycle 37 homolog (S. cerevisiae)-like 1	0.054
rs719725	9q24	Tumor	NM_002839	*PTPRD*	protein tyrosine phosphatase, receptor type, D	0.025
rs10795668	10p14	Tumor	NM_001001973	*ATP5C1*	ATP synthase, H+ transporting, mitochondrial F1 complex, gamma polypeptide 1	0.005
rs3802842	11q23	Tumor	NM_001562	*IL18*	interleukin 18 (interferon-gamma-inducing factor)	0.053
rs3802842	11q23	Tumor	NM_015191	*SIK2*	salt-inducible kinase 2	0.027
rs7136702	12q13	Tumor	NM_052879	*LARP4*	La ribonucleoprotein domain family, member 4	0.023
rs7136702	12q13	Tumor	NM_182559	*TMPRSS12*	transmembrane protease, serine 12	0.043
rs7136702	12q13	Tumor	NM_005653	*TFCP2*	transcription factor CP2	0.005
rs7136702	12q13	Tumor	NM_007210	*GALNT6*	UDP-N-acetyl-alpha-D-galactosamine:polypeptide N-acetylgalactosaminyltransferase 6	0.016
rs7136702	12q13	Tumor	NM_002284	*KRT86*	keratin 86	0.043
rs7136702	12q13	Tumor	NM_020039	*ACCN2*	amiloride-sensitive cation channel 2, neuronal	0.052
rs4444235	14q22	Tumor	NM_007086	*WDHD1*	WD repeat and HMG-box DNA binding protein 1	0.038
rs4444235	14q22	Tumor	NR_003225	*LGALS3*	lectin, galactoside-binding, soluble, 3	0.028
rs4444235	14q22	Tumor	NM_014750	*DLGAP5*	discs, large (Drosophila) homolog-associated protein 5	0.006
rs4779584	15q13	Normal	NM_018648	*NOP10*	NOP10 ribonucleoprotein homolog (yeast)	0.006
rs4779584	15q13	Tumor	NM_017762	*MTMR10*	myotubularin related protein 10	0.053
rs4779584	15q13	Tumor	NM_018648	*NOP10*	NOP10 ribonucleoprotein homolog (yeast)	0.054
rs9929218	16q22	Normal	NM_133458	*ZFP90*	zinc finger protein 90 homolog (mouse)	0.033
rs9929218	16q22	Normal	NM_013241	*FHOD1*	formin homology 2 domain containing 1	0.030
rs9929218	16q22	Normal	NM_145059	*FUK*	fucokinase	0.007
rs9929218	16q22	Normal	NM_012163	*LRRC29*	leucine rich repeat containing 29	0.005
rs9929218	16q22	Normal	NM_032382	*COG8*	component of oligomeric golgi complex 8	0.032
rs9929218	16q22	Normal	NM_018430	*TSNAXIP1*	translin-associated factor X interacting protein 1	0.036
rs9929218	16q22	Normal	NM_001040667	*HSF4*	heat shock transcription factor 4	0.008
rs9929218	16q22	Normal	NM_138383	*MTSS1L*	metastasis suppressor 1-like	0.038
rs9929218	16q22	Normal	NM_033309	*B3GNT9*	UDP-GlcNAc:betaGal beta-1,3-N-acetylglucosaminyltransferase 9	0.011
rs9929218	16q22	Normal	NM_002801	*PSMB10*	proteasome (prosome, macropain) subunit, beta type, 10	0.025
rs9929218	16q22	Normal	NM_003946	*NOL3*	nucleolar protein 3 (apoptosis repressor with CARD domain)	0.0003
rs9929218	16q22	Normal	NM_018380	*DDX28*	DEAD (Asp-Glu-Ala-Asp) box polypeptide 28 // 16q22.1	0.002
rs9929218	16q22	Normal	NM_020786	*PDP2*	pyruvate dehydrogenase phosphatase isoenzyme 2	0.040
rs9929218	16q22	Tumor	NM_018430	*TSNAXIP1*	translin-associated factor X interacting protein 1	0.046
rs961253	20p12	Normal	NM_001009924	*C20orf30*	chromosome 20 open reading frame 30	0.028
rs4925386	20q13	Normal	BC002531	*C20orf149*	chromosome 20 open reading frame 149	0.011
rs4925386	20q13	Normal	NM_020062	*SLC2A4RG*	SLC2A4 regulator	0.016
rs4925386	20q13	Normal	NM_012469	*PRPF6*	PRP6 pre-mRNA processing factor 6 homolog (S. cerevisiae)	0.036
rs4925386	20q13	Normal	NM_018257	*PCMTD2*	protein-L-isoaspartate (D-aspartate) O-methyltransferase domain containing 2	0.054
rs4925386	20q13	Tumor	NM_144498	*OSBPL2*	oxysterol binding protein-like 2	0.024
rs4925386	20q13	Tumor	NM_020882	*COL20A1*	collagen, type XX, alpha 1	0.052
rs4925386	20q13	Tumor	NM_012384	*GMEB2*	glucocorticoid modulatory element binding protein 2	0.032
rs4925386	20q13	Tumor	NM_032957	*RTEL1*	regulator of telomere elongation helicase 1	0.026
